# *Staphylococcus epidermidis*: Antimicrobial Resistance Profiles of Biofilm-Forming Isolates From Pediatric Bacteremia in Pakistan

**DOI:** 10.1155/jotm/8755082

**Published:** 2025-02-10

**Authors:** Maleeha Nisar, Hazir Rahman, Saghir Ahmad, Tabassum Tabassum, Khalid J. Alzahrani, Fuad M. Alzahrani, Khalaf F. Alsharif

**Affiliations:** ^1^Department of Microbiology, Abdul Wali Khan University Mardan, Mardan, Pakistan; ^2^Department of Clinical Laboratories Sciences, College of Applied Medical Sciences, Taif University, P.O. Box 11099, Taif 21944, Saudi Arabia

**Keywords:** biofilm production, *blaZ.*, *mecA*, *msrA*, pediatric bacteremia, *S. epidermidis*

## Abstract

**Background:** *Staphylococcus epidermidis* is an important cause of nosocomial infections in children. The study undertaken identified antibiotic resistance markers among biofilm-forming *S. epidermidis.*

**Methods:** A total of 105 bacteremia-positive samples from hospitalized children were processed for identification of *S. epidermidis* using species-specific *rdr* gene. Phenotypic antibiotic resistance was checked through Kirby–Bauer disc diffusion method. 96-well microtiter plate assays and PCR were used for biofilm production and antibiotic-resistant genes, respectively.

**Results:** Among 105 clinical isolates, *rdr* gene was detected in 34 (32.38%) isolates. The *rdr* detected isolates exhibited biofilm formation (*n* = 34; 100%). Multidrug-resistant (MDR) pattern was observed among *S. epidermidis*, while the frequency of MDR was higher in very strong biofilm-forming *S. epidermidis* (*n* = 18; 52.9%, *p* ≤ 0.002) as compared to weak biofilm-forming *S. epidermidis* (*n = *6; 17.6%). All *S. epidermidis* strains were resistant to cefoxitin, penicillin, and augmentin (*n* = 34; 100%). High resistance was observed against erythromycin (*n* = 29; 85.29%) and ciprofloxacin (*n* = 25; 73.5%). *S. epidermidis* displayed complete susceptibility (*n* = 34; 100%) toward vancomycin, tetracycline, and linezolid. Among the *S. epidermidis* isolates, the methicillin resistance gene (*mecA*, *n* = 29; 85.2%, *p* ≤ 0.000), the erythromycin resistance gene (*msrA*, *n* = 19; 55.7%) and the beta-lactamase resistance gene (*blaZ*, *n* = 17; 50%) were detected. Detection of *mecA* (*n* = 17; 94.4%), *msrA* (*n* = 8; 44.4%) and *blaZ* (*n* = 11; 61.1%) significantly (*p* ≤ 0.0052) correlated with very strong biofilm-forming *S. epidermidis*.

**Conclusion:** Biofilm formation is significantly associated with antibiotic resistance. The study's result will help to understand the molecular mechanism of antimicrobial resistance in biofilm-forming *S. epidermidis* among pediatric patients.

## 1. Introduction

*Staphylococcus epidermidis* is a significant opportunistic pathogen and commonly colonizes the axillae, head, and nares. It has emerged as the common bacteria that forms biofilms, and it is believed to have an essential role in hospital-acquired infections using indwelling devices [1].

It is well documented that disease caused by *S. epidermidis* is due to its ability to form biofilms and avoid the human immune system [2]. For instance, various *S. epidermidis* strains adhere to prosthetic joints, intravascular devices, cerebral fluid shunts, intraocular lenses, and heart valve replacements. Subacute or chronic diseases might result from the pathogen colonization of medical equipment. As a crucial component of patient care, it is recommended to eliminate *S. epidermidis*-induced bacteremia, usually caused by the bacteria getting into the body through contaminated intravascular medical devices [3]. Many *S. epidermidis* hospital strains have a high incidence of multidrug resistance (MDR) (70%–85%), making vancomycin infusion one of the few therapeutic options available [1]. Studies emphasize developing new strategies to treat these infections [4, 5].

Previous studies showed that MDR strains of *S. epidermidis* are present in many newborns admitted to critical care units, and many of these babies die from illnesses brought on by these colonized strains [6, 7]. Furthermore, receiving antibiotic medication for these conditions does not necessarily ensure that these strains will stop colonizing the body [8]. Bacteremia caused by *S. epidermidis* is difficult to treat, and it is associated with longer hospital stays, more healthcare costs, and a significant morbidity rate [9].

In the current study, antibiotic resistance markers of biofilm-forming *S. epidermidis* isolated from pediatric bacteremia were evaluated. Findings of the study will help to understand the mechanism of resistance in biofilm-forming *S. epidermidis*.

## 2. Materials and Methods

### 2.1. Sample Collection

Blood samples were collected from children admitted to a tertiary care hospital (0–9 years). Patient consent was obtained. Ethical approval was granted by the Research Ethics Committee of the Rehman Medical Institute (RMI/RMI-REC/Approval/8, dated December 08, 2020).

### 2.2. Bacterial Isolation and Identification

Bacterial isolation was performed by culturing the samples on blood agar (Thermo Scientific, R01200, USA). Bacterial identification was carried out using morphological and biochemical methods.

### 2.3. DNA Extraction

DNA extraction was done through Chelex-100 (Bio Rad, 142–1253, USA). Colonies from pure cultures were transferred, and Chelex-100 was added to each tube. After adding Chelex-100 to the samples, all tubes were placed in a thermomixer (Eppendorf, 5384000, Germany) at 400 rpm for 30 min at 37°C. After the thermo-mixing, samples were centrifuged at 15,000 rpm for 15 min [10].

### 2.4. Molecular Identification of *S. epidermidis*

Species-specific *rdr* gene primers (forward primer: 5′-AAGAGCGTGGAGAAAAGTATCAAG-3′; reverse primer: 5′-TCGATACCATCAAAAGTTGG-3′). PCR mix (Thermo Scientific, K1081, USA) and thermal conditions for gene amplification were the same as previously described [10].

### 2.5. The 96-Well Microtiter Plate Assay for Biofilm Production

Biofilm-forming ability of *S. epidermidis* isolates was checked by using the 96-well plate (Thermo Scientific, 243656, USA) as previously described [11]. Briefly, the microtiter plate was used to quantify the formation of biofilms. *S. epidermidis* isolates were inoculated in nutrient broth and incubated overnight at 37°C. After diluting (1:100) the cultures, 200 μL was added to a 96-well microtiter plate (Thermo Scientific, 242404, USA). After incubation for 48 h, plates were washed 3 times with 300 μL distilled water and stained with 0.2% crystal violet dye for 10 min, followed by gentle washing with distilled water. The plate was subjected to heat fixation at 60°C for 30 min, followed by the addition of 200 μL acetic acid (33%). The plate was kept in an ELISA reader (BioTek Instruments, ELx800, USA) to note optical densities of all wells at 570 nm. O.D cut-off values were adjusted as; OD ≤ ODC for biofilm negative, ODC < OD ≤ 2 (ODC) for biofilm weak positive, 2 (ODC) < OD ≤ 4 (ODC) for biofilm moderate positive, 4 (ODC) < OD ≤ 5 (ODC) to 4 (ODC) < OD ≤ 9 (ODC) for biofilm strong positive, and 9 (ODC) < OD for very strong positive [10]. Three biological replicates were run to calculate the average OD at 570 nm.

### 2.6. Antimicrobial Susceptibility Testing

The disc-diffusion technique on Muller–Hinton agar (BD Difco, 225250, USA) was used to test the antimicrobial susceptibility of *S. epidermidis* isolates. Penicillin (P, 30 μg), augmentin (AUG, 30 μg), vancomycin (VA, 30 μg), cefoxitin (FOX, 30 μg), gentamicin (CN, 10 μg), erythromycin (E, 15 μg), linezolid (LIN, 30 μg), tetracycline (TE, 30 μg), and ciprofloxacin (CIP, 5 μg) discs (Oxoid, UK) were used. Clinical Laboratory Standard Institute Standard-2021 (CLSI-2021) was used to interpret the zone diameter.

### 2.7. Detection of Antibiotic-Resistant Genes in *S. epidermidis*

Selected antibiotic-resistant genes were amplified (Kyratec, SC300 G-R2, Australia). Briefly, methicillin-resistant gene (*mecA*, forward primer: 5′-AAAATCGATGGTAAAGGTTGGC-3′; reverse primer: 5′-AGTTCTGCAGTACCGGATTTGC-3′), the erythromycin resistance gene (*msrA*, forward primer: 5′-GGCACAATAAAGTGTTTAAAGG-3′; reverse primer: 5′-GGCACAATAAAGTGTTTAAAGG-3′) and the beta-lactamase resistance gene (*blaZ*, forward primer: 5′-ACTTCAACACCTGCTGCTTTC-3´; reverse primer: 5′-TGACCACTTTTATCAGCAACC-3′) were amplified [12–14]. Master mix (Thermo Scientific, K1081, USA) and thermal conditions were used and optimized as previously described [10].

### 2.8. Agarose Gel Electrophoresis

The amplified product was run on 1.5% agarose (Thermo Scientific, 16500100, USA) gel electrophoresis and visualized on UV trans-illuminator (Analytik Jena, 846-03520-2, Germany).

### 2.9. Data Analysis

Data was analyzed using GraphPad software. The Student's *t*-test was used to determine the level of significance. *p* ≤ 0.05 was considered statistically significant.

## 3. Results

**Figure 1:**
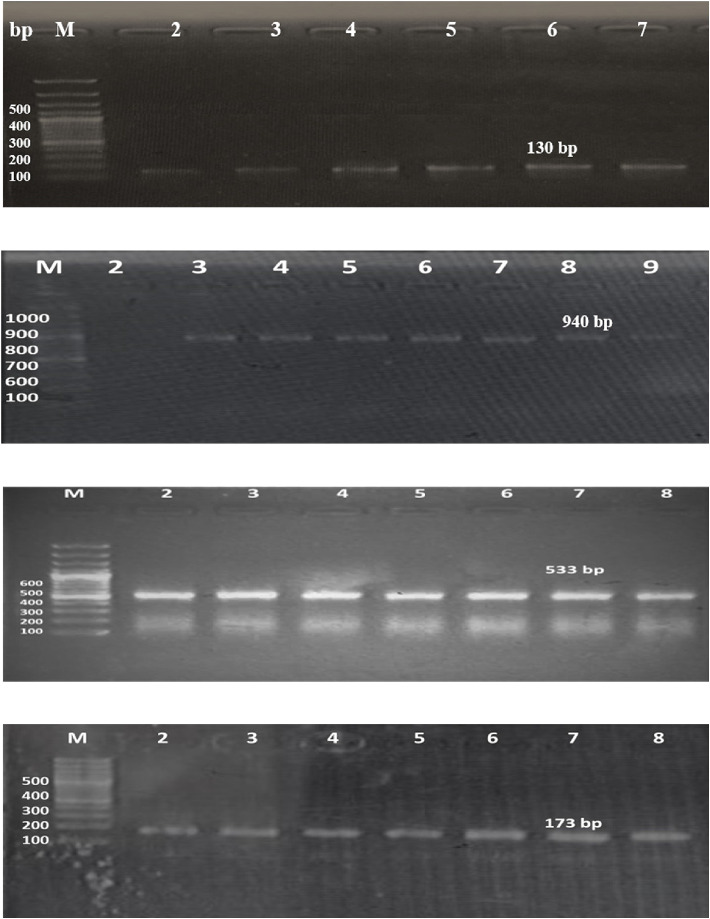
Molecular detection of *S. epidermidis* and profile of resistance markers; (a) amplification of *rdr* (130 bp) for detection of *S. epidermidis*, M: ladder, 2: positive control (molecularly identified *rdr*-positive reference strains of *S. epidermidis* confirmed through mass-spectrometry), 3, 4, 5, 6, 7: *rdr* positive. (b) Amplification of *msfantibiorA* (940 bp), M: ladder, 2: negative control, 3: positive control, 4, 5, 6, 7, 8, 9: *msrA* positive (c) amplification of *mecA* (533 bp), M: ladder, 2: positive control, 3, 4, 5, 6, 7: *mecA* positive. (d) Amplification of *blaZ* (173 bp), M: ladder, 2: positive control, 3, 4, 5, 6, 7, 8: *blaZ* positive.

A total of 105 pediatric bacteremia samples were included in the study. Using molecular methods, 34 (32.3%) isolates were detected as *S. epidermidis* (Figure 1(a)).

**Figure 2:**
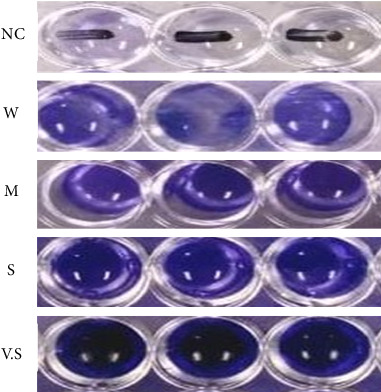
Biofilm-forming ability of isolates on the 96-well microtitre plate assay; NC = negative control having OD = 0.063. W = weak biofilm-forming OD = up to 0.126, I = intermediate biofilm-forming OD = up to 0.252, S = strong biofilm-forming OD = up to 0.378, V.S = very strong biofilm-forming OD > 0.378.

Using the 96-well plate assay, the biofilm-forming ability of each isolate was checked. The results indicated that among the 34 isolates, 6 (17.6%) isolates produced weak biofilm, 3 (8.8%) isolates produced moderate biofilms, 7 (20.7%) isolates produced strong biofilms, while 18 (52.9%) were very strong biofilm producers (Figure 2).

**Figure 3:**
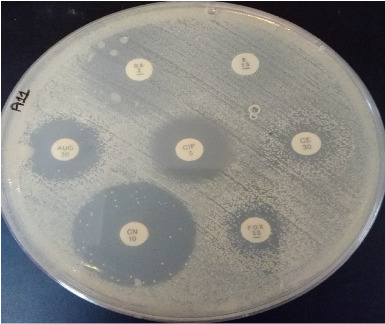
Phenotypic antimicrobial sensitivity of biofilm forming *S. epidermidis.*

After the confirmation of biofilm forming strains, the antibiotic susceptibility profile of these isolates was checked using different antibiotics (Figure 3). The antibiogram of isolates showed significant resistance (*n* = 34; 100%) toward cefoxitin, penicillin, and augmentin. Increased resistance was observed against erythromycin (*n* = 29; 85.29%) and ciprofloxacin (*n* = 25; 73.5%). The sensitivity (*n* = 34; 100%) of *S. epidermidis* was the same for vancomycin, tetracycline, and linezolid. It was noted that antibiotic resistance significantly correlated with biofilm production (Table 1).

Further antibiotic-resistant genes, including *mecA, msrA*, and *blaZ* were detected in *S. epidermidis* isolates through polymerase chain reaction. *MecA* was detected in 29 (85.2%) isolates, *msrA* was detected in 19 (55.8%) isolates, and *blaZ* was detected in 17 (50%) isolates (Figures 1(b), 1(c), 1(d)).

**Figure 4:**
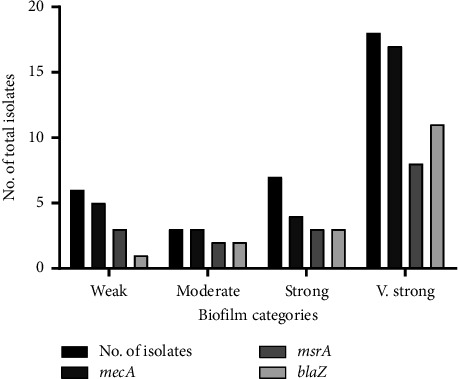
Antibiotic resistance genes (ARGs) among biofilm-forming *S. epidermidis.*

It was noted that the frequency of MDR was higher in very strong biofilm-forming *S. epidermidis* (*n* = 18; 52.9%, *p* ≤ 0.002) as compared to weak biofilm-forming *S. epidermidis* (*n = *6; 17.6%). The resistance genes were also highly prevalent among strong biofilm-forming isolates (Figure 4, Tables 2 and 3).

## 4. Discussion

*S. epidermidis* is a part of the human skin's normal flora [15]. It is sometimes mistakenly thought to be a culture contaminant, even though it may cause several serious ailments [16]. Due to recent developments, it is now a well-known and significant opportunistic pathogen that may cause antibiotic resistance and nosocomial infections such as endocarditis and prosthetic joints infection [17].

Hospital-acquired infections are frequently caused by *S. epidermidis*. Most of these infections are caused by biofilm growing on indwelling medical equipment. The frequent asymptomatic biofilm-associated infections are caused by *S. epidermidis* [18]. Among the molecular components that contribute to the colonization, adhesion, and other biofilm-forming activities that enable *S. epidermidis* infection to be successful are genes encoding polysaccharides and proteins [19]. Antibiotic resistance manifests as biofilm development, which complicates the treatment of illnesses caused by medical devices that foster the growth of antibiotic-resistant microbes.

In the current study, 34 clinical isolates of *S. epidermidis* that were taken from hospitalized children were shown to be resistant toward ciprofloxacin, augmentin, cefoxitin, erythromycin, and penicillin. The Staphylococcal cassette chromosome (SCC), one of the most significant mobile genetic components in staphylococci, is essential for the transmission of methicillin resistance genes from one strain to another [20]. In contrast to a previous study [21], which reported 86 methicillin-resistant *S. epidermidis* clinical isolates, our investigation demonstrates 100% methicillin-resistant *S. epidermidis*. Hospitals have been reported to have high rates of methicillin resistance. This may be related to the regular use of beta-lactam antibiotic, which may lead to the development of resistant bacteria and their subsequent dissemination across the community [22].

Hospital isolates of *S. epidermidis* showed 85% resistance to erythromycin. Comparable results were found in another investigation [21] that found 82% of *S. epidermidis* isolated from patients were resistant to erythromycin. One study from Poland reported that 44% *S. epidermidis* isolates in hospital ambient were resistant to erythromycin [22]. 25 (73.5%) isolates were resistant toward ciprofloxacin in our study, while 81% ciprofloxacin-resistance in hospital isolates were reported by another study [23].

In the current study, 29 (85.2%) isolates were *mecA* positive. Another study [24] found that 27(87%) of the 31 clinical isolates of *S. epidermidis* had a similar *mecA* frequency. In another investigation [25], 89% of clinical isolates of *S. epidermidis* were shown to be *mecA* positive. Out of 34 isolates, 19 (55.8%) were *msrA* positive. The *msrA* gene is 59% prevalent in clinical isolates, according to a similar study conducted on hospital isolates [26]. Conversely, another study [25] revealed a 53% prevalence of *msrA* in clinical isolates. Among 34 samples, the study detected the *blaZ* gene in 17 (50%) *S. epidermidis* isolates, lower than detected by another finding (*n* = 80; 80%) indicating variations in the encoding of beta-lactamase in Staphylococci [14]. The prevalence of antibiotic resistance genes *mecA, msrA* and *blaZ* in biofilm-forming isolates showed that the most prevalent antibiotic-resistant genes were mainly found in strong biofilm-forming strains as compared to weak biofilm producers.

Analysis of the 34 isolates revealed that 18 (52.9%) isolates were very strong biofilm producers, while 7 (20.5%), 3 (8.8%), and 6 (17.6%) isolates were strong, intermediate, and weak biofilm-forming, respectively. The antibiotic resistance pattern based on biofilm forming ability showed that all strong biofilm-forming isolates showed 100% resistance against penicillin, augmentin, and cefoxitin. Similar findings were obtained by two previous studies, where 97% methicillin resistance was detected in clinical *S. epidermidis* that were frequently forming biofilms (86.6%) [10, 27]. It shows that biofilm formation and antibiotic resistance are factors commonly present in *S. epidermidis* of clinical origin.

Biofilms are complex structures having intrinsic acquisition of antimicrobial resistance (AMR). Glycocalyx formation, an integral part of microbial biofilms, accumulates 25% of an antimicrobial drug, hence limiting deep diffusion [28]. The enzyme mediated resistance in biofilms converts a biocidal molecule to its non-toxic form [29]. Furthermore, metabolic heterogeneity of biofilms, appearance of persister cells, development of biofilm-specific growth behavior, genetic adaptation for distinct protective phenotypes, quorum-sensing, stress responses [30], expression of efflux pumps, and MDR-operons in biofilms state by suboptimum concentrations of antimicrobials [31, 32] are the intrinsic and acquired antibiotic-resistant mechanisms in mature biofilms. It is worth noting that the virulence potential (biofilm formation) of *S. epidermidis* was significantly associated with MDR. Isolates forming very strong biofilms were significantly resistant to beta-lactams including penicillin, augmentin and cefoxitin. *S. epidermidis* frequently produces strong biofilms while significantly encoding-*mecA*. *mecA* encoding methicillin resistance was significantly associated with very strong biofilm-positive *S. epidermidis* (*n* = 29; 85.2%, *p*=0.005). Antibiotic resistance genes are rapidly transmitted in biofilm states of growth compared to planktonic lifestyle [33], which might further increase antibiotic resistance and create serious concerns regarding the use of beta-lactam antibiotic in treatments of pediatric bacteremia caused by biofilm-forming *S. epidermidis*.

### 4.1. Limitations

This study is a single-center experience; however, studies from multiple centers are recommended to check the association of biofilm formation and antibiotic resistance simultaneously present in *S. epidermidis*. As the current study individually checked *S. epidermidis* encoding antibiotic resistance and forming strong biofilms, studies are recommended to directly check the antibiotic resistance potential of *S. epidermidis* during biofilm formation.

## 5. Conclusion

Biofilm formation among *S. epidermidis* isolates in pediatric bacteremia was significantly associated with antibiotic resistance. Findings of the study will help in understanding the role of biofilm production in the development of AMR in *S. epidermidis* causing children bacteremia.

## Figures and Tables

**Table 1:** Antibiotic susceptibility of biofilm forming *S. epidermidis* isolates from pediatric bacteremia.

Antibiotic	Overall		Biofilm status											
			Very strong (*n* = 18)			Strong (*n* = 7)			Moderate (*n* = 3)			Weak (*n* = 6)		
	Susceptible *n* (%)	Resistant *n* (%)	Susceptible *n* (%)	Resistant *n* (%)	*p* value	Susceptible *n* (%)	Resistant *n* (%)	*p* value	Susceptible *n* (%)	Resistant *n* (%)	*p* value	Susceptible *n* (%)	Resistant *n* (%)	*p* value
Methicillin	0 (0)	⁣^∗^34 (100)	0 (0)	18 (100)	≤ 0.001	0 (0)	7 (100)	≤ 0.001	0 (0)	3 (100)	≤ 0.001	0 (0)	6 (100)	≤ 0.001
Augmentin	0 (0)	⁣^∗^34 (100)	0 (0)	18 (100)	≤ 0.001	0 (0)	7 (100)	≤ 0.001	0 (0)	3 (100)	≤ 0.001	0 (0)	6 (100)	≤ 0.001
Penicillin	0 (0)	34 (100)	0 (0)	18 (100)	≤ 0.001	0 (0)	7 (100)	≤ 0.001	0 (0)	3 (100)	≤ 0.001	0 (0)	6 (100)	≤ 0.001
Vancomycin	34 (100)	0 (0)	18 (100)	0 (0)	≤ 0.001	7 (100)	0 (0)	≤ 0.001	6 (100)	0 (0)	≤ 0.001	3 (100)	0 (0)	≤ 0.001
Gentamicin	23 (67.64)	11 (32.35)	12 (66.6)	6 (33.3)	≤ 0.05	4 (57.1)	3 (42.8)	≥ 0.05	0 (0)	3 (100)	≤ 0.001	3 (50)	3 (50)	≥ 0.05
Erythromycin	5 (14.71)	⁣^∗^29 (85.29)	4 (22.2)	14 (77.7)	≤ 0.05	0 (0)	7 (100)	≤ 0.001	0 (0)	3 (100)	≤ 0.001	1 (16.6)	5 (83.3)	≤ 0.05
Tetracycline	34 (100)	0 (0)	18 (100)	0 (0)	≤ 0.001	7 (100)	0 (0)	≤ 0.001	6 (100)	0 (0)	≤ 0.001	3 (100)	0 (0)	≤ 0.001
Linezolid	34 (100)	0 (0)	18 (100)	0 (0)	≤ 0.001	7 (100)	0 (0)	≤ 0.001	6 (100)	0 (0)	≤ 0.001	3 (100)	0 (0)	≤ 0.001
Ciprofloxacin	9 (37.5)	25 (73.5)	5 (27.7)	13 (72.2)	≤ 0.05	2 (28.5)	5 (71.4)	≥ 0.05	0 (0)	3 (100)	≤ 0.001	2 (33.3)	4 (66.6)	≥ 0.05

⁣^∗^Significant resistance in very strong biofilm-forming *S. epidermidis*, *p* ≤ 0.05 is considered as significant while *p* ≥ 0.05 is considered as non-significant.

**Table 2:** Evaluation of biofilm profile versus antibiotic resistance genes among *S. epidermidis* isolates.

Biofilm categories	MDR isolates *n* (%)	*mecA n* (%)	*msrA n* (%)	*blaZ n* (%)	*p* value
Weak	6 (17.6)	5 (83.3)	3 (100)	1 (16.6)	≥ 0.05
Moderate	3 (8.82)	3 (100)	2 (66.6)	2 (66.6)	≥ 0.05
Strong	7 (20.5)	4 (57.1)	3 (42.8)	3 (42.8)	≥ 0.05
Very strong	18 (52.9)	17 (94.4)	8 (44.4)	11 (61.1)	≤ 0.05
Total	34	29 (85.2)	19 (55.8)	17 (50)	≤ 0.05

⁣^∗^Significant resistance in very strong biofilm-forming *S. epidermidis*, *p* ≤ 0.05 is considered as significant, while *p* ≥ 0.05 is considered as non-significant.

**Table 3:** Overall antibiotic resistance profile of biofilm-forming *S. epidermidis* (*n* = 34).

Sample no.	Biofilm status	Phenotypic markers				Genotypic markers		
		Resistance		Sensitive		*mecA*	*msrA*	*blaZ*
		Antibiotic	*n*	Antibiotic	*n*			
1	V. strong	M, P, AUG, E, CIP	5	VA, LIN, TE, CN	4	Detected	Detected	Not detected
2	Strong	M, P, AUG, E, CIP	5	VA, LIN, TE, CN	4	Detected	Not detected	Detected
3	Weak	M, P, AUG, CN, E	5	VA, LIN, TE, CN, CIP	5	Detected	Not detected	Detected
4	V. strong	M, P, AUG, CN	4	VA, LIN, TE, E, CIP	5	Detected	Not detected	Not detected
5	V. strong	M, P, AUG, E, CIP	5	VA, LIN, TE, CN	4	Detected	Not detected	Detected
6	V. strong	M, P, AUG, E, CIP	5	VA, LIN, TE, CN	4	Detected	Detected	Not detected
7	Strong	M, P, AUG, E, CIP	5	VA, LIN, TE, CN	4	Detected	Not detected	Detected
8	Moderate	M, P, AUG, E, CIP	5	VA, LIN, TE, CN	4	Not detected	Detected	Not detected
9	V. strong	M, P, AUG, E, CIP	5	VA, LIN, TE, CN	4	Detected	Detected	Detected
10	V. strong	M, P, AUG, CIP	4	VA, LIN, TE, E, CN	5	Detected	Not detected	Not detected
11	V. strong	M, P, AUG, E, CIP	5	VA, LIN, TE, CN	4	Detected	Detected	Not detected
12	Weak	M, P, AUG, E, CIP	5	VA, LIN, TE, CN	4	Not detected	Detected	Detected
13	V. strong	M, P, AUG, E, CIP	5	VA, LIN, TE, CN	4	Detected	Detected	Detected
14	V. strong	M, P, AUG, CN, E	5	VA, LIN, TE, CIP	4	Detected	Detected	Not detected
15	V. Strong	M, P, AUG, CN, CIP	5	VA, LIN, TE, E	4	Detected	Not detected	Detected
16	Strong	M, P, AUG, E, CIP	5	VA, LIN, TE, CN	4	Detected	Detected	Not detected
17	V. strong	M, P, AUG, E, CIP	5	VA, LIN, TE, CN	4	Detected	Not detected	Not detected
18	Moderate	M, P, AUG, E, CIP	5	VA, LIN, TE, CN	4	Detected	Detected	Detected
19	V. strong	M, P, AUG, E, CIP	5	VA, LIN, TE, CN	4	Detected	Detected	Not detected
20	V. strong	M, P, AUG, CN, CIP	5	VA, LIN, TE, E	4	Detected	Not detected	Detected
21	V. strong	M, P, AUG, CN, E	5	VA, LIN, TE, CIP	4	Detected	Detected	Not detected
22	Strong	M, P, AUG, CN, E	5	VA, LIN, TE, CIP	4	Detected	Detected	Detected
23	Strong	M, P, AUG, E, CIP	5	VA, LIN, TE, CN	4	Detected	Not detected	Not detected
24	Weak	M, P, AUG, CN, E	5	VA, LIN, TE, CIP	4	Detected	Not detected	Detected
25	Weak	M, P, AUG, E, CIP	5	VA, LIN, TE, CN	4	Detected	Not detected	Not detected
26	Strong	M, P, AUG, CN, E, CIP	6	VA, LIN, TE	3	Not detected	Detected	Detected
27	Strong	M, P, AUG, CN, E	5	VA, LIN, TE, CIP	4	Detected	Not detected	Detected
28	V. strong	M, P, AUG, E, CIP	5	VA, LIN, TE, CN	4	Detected	Detected	Not detected
29	Weak	M, P, AUG, E, CIP	5	VA, LIN, TE	4	Detected	Detected	Detected
30	V. strong	M, P, AUG, E	4	VA, LIN, TE, CN, CIP	4	Detected	Detected	Not detected
31	Moderate	M, P, AUG, E, CIP	5	VA, LIN, TE, CN	4	Not detected	Detected	Not detected
32	V. strong	M, P, AUG, CN, E	5	VA, LIN, TE, CIP	4	Detected	Not detected	Detected
33	V. strong	M, P, AUG, E, CIP	5	VA, LIN, TE, CN	4	Not detected	Detected	Not detected
34	Weak	M, P, AUG, CN, CIP	5	VA, LIN, TE, E	4	Detected	Not detected	Detected

*Note:* CN, gentamicin. “*n*” indicates the number of antibiotic the isolate was resistant or sensitive to.

Abbreviations: AUG, augmentin; CIP, ciprofloxacin; E, erythromycin; LIN, linezolid; M, methicillin; P, penicillin; TE, tetracycline; VA, vancomycin; V. strong, very strong.

## Data Availability

The data that supports the findings of this study are available from the corresponding author upon reasonable request.
